# Selection and Optimization of a Bioink Based on PANC-1- Plasma/Alginate/Methylcellulose for Pancreatic Tumour Modelling

**DOI:** 10.3390/polym15153196

**Published:** 2023-07-27

**Authors:** Cristina Banda Sánchez, Nieves Cubo Mateo, Laura Saldaña, Alba Valdivieso, Julie Earl, Itziar González Gómez, Luis M. Rodríguez-Lorenzo

**Affiliations:** 1 Institute of Science and Technology of Polymers (ICTP-CSIC), 28006 Madrid, Spain; 2 Nebrija Research Group ARIES, Higher Polytechnic School, Antonio de Nebrija University, 28015 Madrid, Spain; 3 Institute for Physical and Information Technologies (ITEFI-CSIC), Sensors and Ultrasonic Systems, 28006 Madrid, Spain; 4 IdiPAZ, Hospital Universitario La Paz, 28046 Madrid, Spain; 5 Biomedical Research Networking Center in Bioengineering, Biomaterials, and Nanomedicine, CIBER-BBN, 28029 Madrid, Spain; 6 Ramón y Cajal Health Research Institute (IRYCIS), Molecular Epidemiology and Predictive Tumour Markers, 28034 Madrid, Spain; 7 Biomedical Research Network in Cancer (CIBERONC), 28034 Madrid, Spain

**Keywords:** 3D bioprinting, bioinks, plasma, alginate, methylcellulose, PANC-1, tumour modelling

## Abstract

3D bioprinting involves using bioinks that combine biological and synthetic materials. The selection of the most appropriate cell-material combination for a specific application is complex, and there is a lack of consensus on the optimal conditions required. Plasma-loaded alginate and alginate/methylcellulose (Alg/MC) inks were chosen to study their viscoelastic behaviour, degree of recovery, gelation kinetics, and cell survival after printing. Selected inks showed a shear thinning behavior from shear rates as low as 0.2 s^−1,^ and the ink composed of 3% *w/v* SA and 9% *w/v* MC was the only one showing a successful stacking and 96% recovery capacity. A 0.5 × 10^6^ PANC-1 cell-laden bioink was extruded with an Inkredible 3D printer (Cellink) through a D = 410 μm tip conical nozzle into 6-well culture plates. Cylindrical constructs were printed and crosslinked with CaCl_2_. Bioinks suffered a 1.845 Pa maximum pressure at the tip that was not deleterious for cellular viability. Cell aggregates can be appreciated for the cut total length observed in confocal microscopy, indicating a good proliferation rate at different heights of the construct, and suggesting the viability of the selected bioink PANC-1/P-Alg_3_/MC_9_ for building up three-dimensional bioprinted pancreatic tumor constructs.

## 1. Introduction

A major hurdle in cancer research is the pre-clinical modelling strategies that have been conventionally applied to evaluate the efficacy of new cancer therapies. Traditionally, new anticancer drugs have been evaluated in two-dimensional (2D) cell culture platforms. However, 2D cultured cancer cells cannot mimic the complexity and heterogeneity of in vivo tumours, which usually grow in a three-dimensional (3D) conformation [1]. During the last few years, several 3D cell culturing techniques have emerged to overcome the observed gap between in vivo and in vitro experiments in cancer research [2]. Three-dimensional cancer models are anticipated to mimic the in vivo tumour microenvironment in human patients by recapitulating the proper tumour cell/matrix composition and yielding properties that match the type and stage of the intended disease. Therefore, it would be possible to perform accurate mechanistic studies on these in vitro models [3]. Many of these techniques base their performance on the generation of multicellular tumour spheroids (MCTS) [4], hydrogel embedding [5], cell patterning [6], or microfluidic chips [7]. These techniques are helpful for many studies. i.e., multicellular tumour spheroids have been used to study fundamental cancer biology and drug screening [8,9]. However, the tumour microenvironment, including both chemical cues (growth factors and cytokines) and biophysical cues (interstitial pressure and matrix mechanics), is extremely complex, and most of those models lack well-organized spatial distribution of tumour cells and ECM compositions [3]. Therefore, there are still significant challenges that need to be overcome by 3D in vitro models on the road to using them for therapeutic drug development. These include batch-to-batch variability [10], limited control over cell patterning [11], low throughput, oversimplified structures [3] and limited vascularization potential [12].

3D bioprinting combines the ability to design geometrical parameters for constructs such as pore size, pore strut thickness, pore interconnectivity, and pores morphology with the capacity of depositing several types of co-cultured cells in a single spatial arrangement matching the natural architecture of native tissues [13,14]. This tissue engineering approach may be reproduced to engineer preclinical tumour models resulting in spatiotemporal control of physical and biological elements [15]. Mechanical forces, different cell populations [16] and bioactive signals can be incorporated into biological environments [17] to drive cell phenotypes that resemble tumour microenvironments [18]. 3D bioprinting involves using bioinks that combine biological and hosting synthetic materials. The selection of the most appropriate cell-material combination for a specific application, the printing conditions (printer type, temperature oxygen rate, speed of deposition), and the maturation procedure (signals and bioreactors) are so complex that there is a lack of consensus in the optimal conditions required for each specific case [19]. In addition, when tumour modelling is considered, there is high variability between cancer types and stages, making the selection of conditions even more specific.

Developing suitable cell-material combinations that can be used as bioinks for each specific application is the bottleneck hindering the advance of bioprinting. In addition to the already strict conditions that made a material suitable for building up a cytocompatibility scaffold, there are very specific conditions that materials should fulfil to be used as components in bioinks. Alginate has been selected as the first component of the intended bioink due to its gelling capacity, low toxicity, high availability, and low cost. Alginate hydrogels are formed by ionic crosslinking in the presence of calcium ions, and they reach similar mechanical properties to extracellular matrices, which explains why they are frequently used for studying cell response in tissue engineering testing. In addition, alginates have been declared safe by the United States Food and Drug Administration (FDA) [20] for application in humans [21]. Literature search results in terms of cell viability for different alginate concentrations [22] and previous works from team members [23] were used to select a 3% *w*/*v* alginate concentration as starting reference. The combination of viscoelastic values in the ECM range, potential cell viability and ease of preparing/handling was the criteria used. However, building up 3D printed construct in the Z direction with alginate hydrogels has been described as non-successful [24], whereas blending with a second polysaccharide, such as methylcellulose (MC), makes it easier to modulate the viscoelastic properties of the material and enables the printing of strands in the Z direction without fusing [25]. MC is a water-soluble cellulose derivative, cytocompatible and approved for food and drug administration [24].

This manuscript intends to describe the selection of a bioink (cell-material combination) that can be used in the future in modelling pancreatic tumours. Plasma was incorporated not only because plasma-based bioinks have demonstrated that they allow cell spreading, colony formation and angiogenic cues even for long-term in vitro tests [23]. In addition, plasma contains fibrinogen that should help induce fibroblast to generate the thick fibrous walls characteristic of pancreatic tumours when multicellular models are assayed [26]. Pancreatic tumour cells, PANC-1, have been shown to grow well in vitro 2D as attached cells [27] and were selected for this proof-of-concept study.

This paper deals with the translation and validation of a recently proposed synthetic material composition in the field of bone engineering [23] for preparing a PANC-1-based bioink for future modelling of primary pancreas tumours and with the establishing of the procedure and conditions required for the validation of a bioink. The states that must be established include viscoelasticity properties, gelation characteristics, geometrical fidelity and cellular viability after the stress suffered by the selected cell line within the tip during the bioprinting process.

## 2. Materials and Methods

### 2.1. Materials and Cells

#### 2.1.1. Polymers

Alginic acid sodium salt from brown algae (SA) (Mw: 8945 g/mol, mannuronate/guluronate ratio of 0.63, Sigma, Saint-Quentin-Fallavier, France) [28] and methylcellulose (MC) (viscosity 4000 cP, M0512-500G, Sigma were purchased by Sigma-Aldrich (Madrid, Spain). Fresh frozen human plasma was donated by Hospital Ciudad de Coria. In this study, fibrinogen concentration was not measured, but coagulation was tested through a tube inverted test, obtaining a coagulation time below 10 min. For the inverted tube test, three vials were filled with 1 mL of plasma, and then 0.2 mL of CaCl_2,_ 1.5% *w*/*v,* was added while a timer was started for each one. Tube inversion was performed every 30 s to visually inspect the sol-gel transition until the gel remained attached to the top. To have a reference, as previously reported in the literature, fibrinogen’s normal concentration range is 200 to 400 mg/dL (2.0 to 4.0 g/L), which may vary slightly among different laboratories and patients [29].

#### 2.1.2. Cells

PANC-1 cells, an epithelioid carcinoma cell line derived from the human pancreas [30], were provided by ATCC (frozen, CRL-1469, tissue: Pancreas; Duct) from the human cell culture collection (https://www.atcc.org/ accessed on 1 September 2020).

### 2.2. Inks and Bioink Preparation

Fresh frozen human plasma was slowly thawed at 37 °C and mixed with SA under stirring. Then, MC was added and mixed with a spatula. Three different inks were prepared using the following concentrations: plasma containing alginate 3% *w/v* (P-Alg_3_/MC_0_), plasma containing alginate 3% *w/v*, methylcellulose 3% *w/v* (P-Alg_3_/MC_3_) and plasma containing alginate 3% *w/v*, methylcellulose 9% *w/v* (P-Alg_3_/MC_9_).

For the bioink (PANC-1/P-Alg_3_MC_9_), standard cell culture protocols for PANC-1 cell lines were applied for cell sample preparation [30,31]. PANC-1 cell lines were cultured in RPMI (Gibco/Invitrogen Bleiswijk, Netherlands) supplemented with 10% fetal bovine serum (FBS; Invitrogen Bleiswijk, Netherlands) and 50 units/mL penicillin/streptomycin (Invitrogen) and kept in an incubator at 5% CO_2_ and 37 °C. Then cells were resuspended and embedded into the P-Alg_3_/MC_9_ ink, slowly mixing with a spatula, obtaining a final concentration of 5 × 10^5^ cells/mL. Every procedure is accomplished under sterile conditions inside a laminar flow cabinet.

### 2.3. Optimization & Characterization of the Inks

Inks printability was assessed by a layer stacking test, printing 20 mm successive layers with a pneumatic extrusion Inkredible 3D printer (Cellink) through a conical nozzle (Nordson, SmoothFlow Tapered Dispense Tips, ref 7018298, ID = 0.41 mm/0.016″) and visually analyzing the merge of the layers. Also, rheological tests were performed on an ARG2 (TA instruments, New Castle, DE, USA) rheometer with a sandblasted parallel plate D = 25 mm geometry at 37 °C before, during, and after the gelation event upon the addition of 1.5% *w/v* CaCl_2_ crosslinker [32]. From a parallel plate geometry, the Power law index (η) (Equation (1)) is obtained as previously described [33]:

(1) $$\eta =k{\ddot{\Upsilon }}^{n-1}$$

where η is viscosity, k is the consistency and ϔ the shear rate. Oscillatory frequency sweeps evaluate the viscosity behaviour at the shear thinning region. The linear viscoelastic region had a torque sweep between 0.5 and 10^3^ s^−1^. The critical yield point, γc = 154 μNm, determined from the LVR test, delimits the maximum value of stress that the ink can support. Frequency tests were made below that value. Frequency sweeps, in the range from 10^−2^ to 10^2^ Hz, were performed at a torque of 50 μNm. Thixotropy and degree of recovery after a structural disruption are computed by applying a low shear rate (0.5 s^−1^) for 60 s and then a 250 s^−1^ shear for 30 s. Gelation kinetics are measured in a time sweep for 30 min, where crosslinker (1.5% CaCl_2_) is added 1 min after starting recording. All rheological treatments have been replicated three times.

### 2.4. 3D Bioprinting

Cell-laden bioink solution is loaded into a 5 mL cartridge and then stored at RT to stabilise bioink’s rheological properties. Pneumatic extrusion Inkredible 3D printer (Cellink) dispenses the hydrogel bioink through a conical nozzle (D = 410 μm). Cylindrical constructs of D = 15 mm are printed into 6-well cell culture plates with a strand width of 1.8 mm, creating 3D stacking pores of 1.3 mm (with a 0.48 mm infill extrusion width). Printed hydrogels are crosslinked for 20 min under a bath with 1.5% *w/v* CaCl_2_; then, the crosslinker is removed and replaced with fresh medium. Finally, constructs are incubated at 37 °C, 5% CO_2_.

Shear stress suffered by the bioinks in the syringe is calculated with the equation.

(2) $${\dot{\gamma }}_{1}=\left(\frac{V_{1}R_{1}^{2}}{\left(\frac{n}{3n+1}\right)\left(R_{1}^{\frac{3n+1}{n}}\right)}\right)\sqrt[{n}]{r}$$

And the shear stress suffered at the tip is calculated as shown in Equation (3):

(3) $${\dot{\gamma }}_{2}=\left(\frac{V_{2}R_{2}^{2}}{\left(\frac{n}{3n+1}\right)\left(R_{2}^{\frac{3n+1}{n}}\right)}\right)\sqrt[{n}]{r}$$

where $\dot{\gamma }$ is the shear rate, *V* is the dispensing speed, *R* is the maximum ratio, and *r* is the ratio at the calculation point.

### 2.5. Viability Assay on Cell-Laden Constructs

Cellular survival and proliferation are assessed with a Live/Dead viability kit, calcein AM (0.5 mL) and ethidium homodimer-1 (2.0 mL) were dissolved in 997.5 mL PBS, added to the samples, and incubated for 30 min while protected from light at 37 °C in a humidified atmosphere with 5% CO_2_. (EthD-1) (ThermoFisher Scientific #L3224, Karlsruhe, Germany) according to the manufacturer’s protocol. Afterwards, constructs are imaged in a fluorescence microscope (Smart Cell Imager PAULA, Leica Microsystems, Wetzlar, Germany) for green (live) and red (dead) spectra. Viability is screened at 1-, 6-, and 14-day post-printing with a Leica TCS SPE, Wetzlar, Germany, from MNCN-CSIC, Madrid. For the visualization of nuclear morphology and proliferation within the construct, cells were fixed with 4% *w*/*v* formaldehyde in PBS and stained with PBS containing 3 × 10^−6^ M, 4,6-diamidino-2-phenylindole DAPI (D1306, Invitrogen) and Actin (Alexa Fluor 488 phalloidin, A12379, Invitrogen) and by confocal microscopy using a confocal microscope (Leica TCS SPE, Wetzlar, Germany) from idiPAZ-Madrid.

### 2.6. Statistics

Three repetitions have been conducted to verify the reproducibility of the results obtained in the rheology and cytocompatibility test. A One-way ANOVA was performed. Pairwise comparisons of means with equal variances have been implemented using Tukey’s post hoc analysis. Statistical differences were assumed at *p* < 0.05. All analyses were performed using STATA/SE, StataCorp LLC Statistics/Data Analysis (Special Edition College Station, TX 77845, USA).

## 3. Results

### 3.1. Ink Selection, Printability and Printing Fidelity

**Figure 1:**
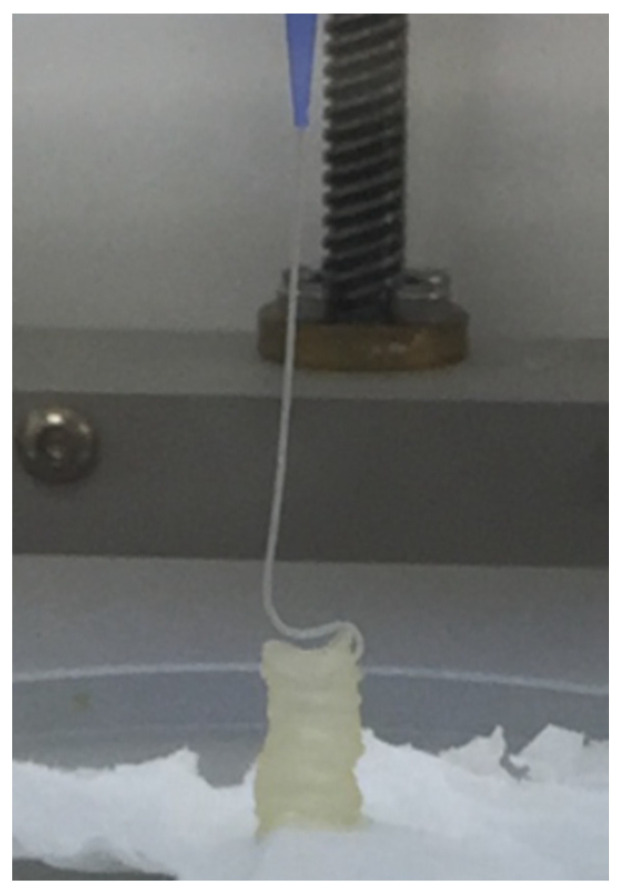
Layer stacking test. Ink containing plasma, 3% alginate and 9% methylcellulose (P-Alg_3_/MC_9_) is the only one where successive layers did not merge with the layers below.

Plasma-based bioinks containing an alginate concentration of 3% and Methyl-cellulose (MC) to refine printability was selected based on published works for applications in bone engineering [23]. Three different concentrations of MC were tested: 0, 3 and 9%. A layer stacking test was used for the initial qualitative screening. It could be observed that a successful stacking without merging of the layers is obtained only for the composition P-Alg_3_/MC_9_, as displayed in Figure 1.

**Figure 2:**
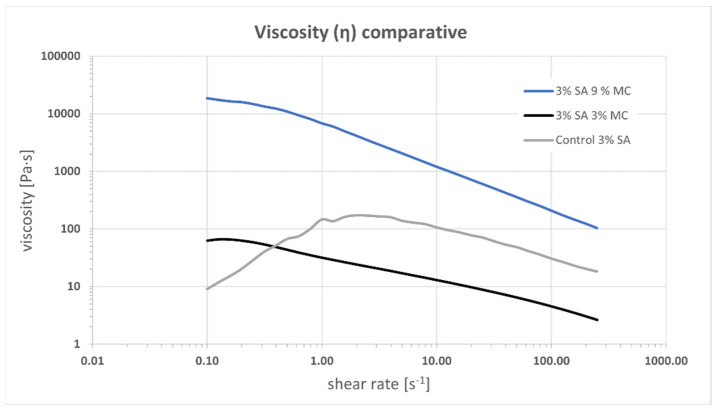
Viscosity vs. shear rate for inks P-Alg_3_/MC_0_, P-Alg_3_/MC_3_ and P-Alg_3_/MC_9._ An extended shear thinning region can be observed for MC-containing inks.

Printability was evaluated, then, through rheology tests. Viscosity versus shear rate curves are shown in Figure 2. They are obtained using the Cox-Merz transformation. The two MC-containing inks, P-Alg_3_/MC_3_ and P-Alg_3_/MC_9_ show a shear thinning behaviour from a yield stress point as low as 0.2 s^−1^. Whereas the control ink, P-Alg_3_/MC_0_, shows a shear thinning behaviour from a yield stress point of 2 s^−1^, indicating that the inclusion of the MC expands the range of shear rate that can be applied when printing. Also, a greater viscosity is obtained for P-Alg_3_/MC_9_. The n Index, between 0.2–0.3, for P-Alg_3_/MC_3_ and P-Alg_3_/MC_9_ indicates a rapid decrease of viscosity with the increase of shear rate that it should favour not only the printability but also the recovery of the viscosity of the ink when the printing process is over.

**Figure 3:**
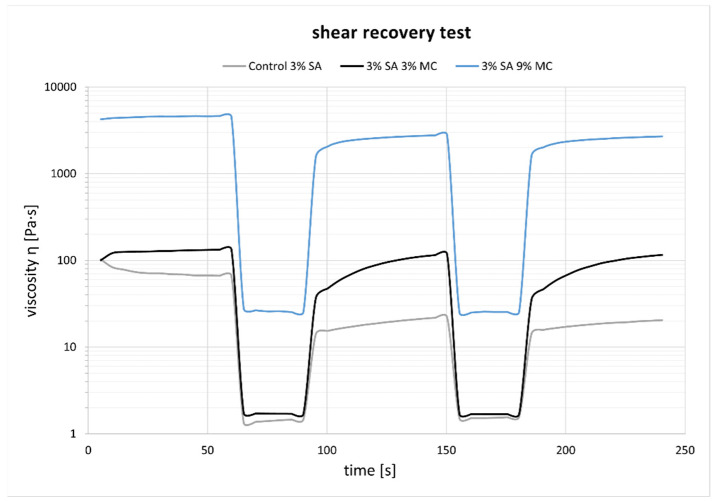
Recovery test experiments. P-Alg_3_/MC_9_ displays the highest and quickest recovery percentage out of the three tested inks.

The recovery experiments for the three tested inks are shown in Figure 3. P-Alg_3_/MC_9_ ink eventually recovers 96% of the viscosity after the period of high shear rate, whereas P-Alg_3_/MC_3_ recovers 85% and P-Alg_3_/MC_0_ recovers 89% in the first cycle, but it drops in the second. Also, P-Alg_3_/MC_9_ displays a quicker recovery in the first 60 s after disruptive shear stress application than P-Alg_3_/MC_3._

**Figure 4:**
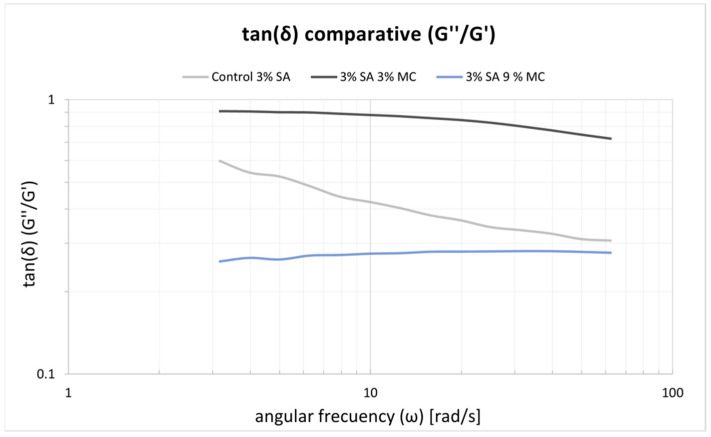
tan(δ) results obtained with the frequency swaps for the three inks. P-Alg_3_/MC_9_ is stable over the whole range of frequencies.

The frequency sweeps made on the inks are shown in Figure 4. Similar rubbery behaviour is observed for the three inks along the tested range of frequencies where the elastic behaviour prevails. P-Alg_3_/MC_0_ displays a fall in tan(δ) for higher frequencies. Similar behaviour is also observed for P-Alg_3_/MC_3_. However, this ink is more stable at low frequencies, and P-Alg_3_/MC_9_ is stable over the whole range of frequencies, also showing the lower value for tan(δ), indicating the highest elastic vs. viscous behaviour of the three inks. Thus, the highest capacity for absorbing impacts with no deformation.

**Figure 5:**
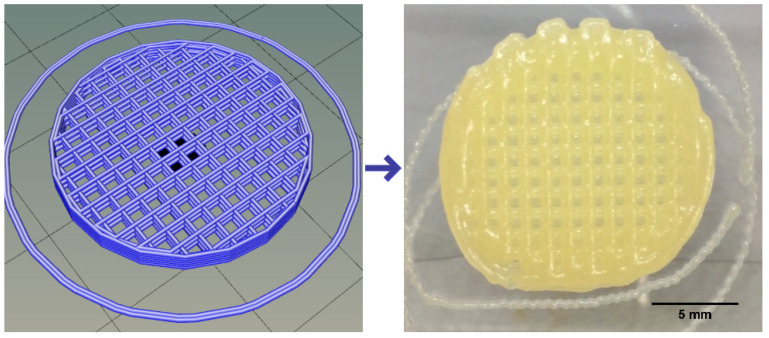
CAD design on the left and printed device on the right reproducing the design.

From these results, P-Alg_3_/MC_9_ was chosen for printing PANC-1-loaded models. Printing fidelity was then evaluated to learn the capacity of this ink to reproduce accurately the design. The CAD design and the printed result with P-Alg_3_/MC_9_ ink can be shown in Figure 5. At the time of printing, the ink offers great printability in terms of layer stacking, shape consistency and CAD fidelity, no ink clumping at the tip of the nozzle, and homogeneous qualitative ink distribution. The external skirt has been used to determine the average required printing pressure of 78 ± 1 kPa.

**Figure 6:**
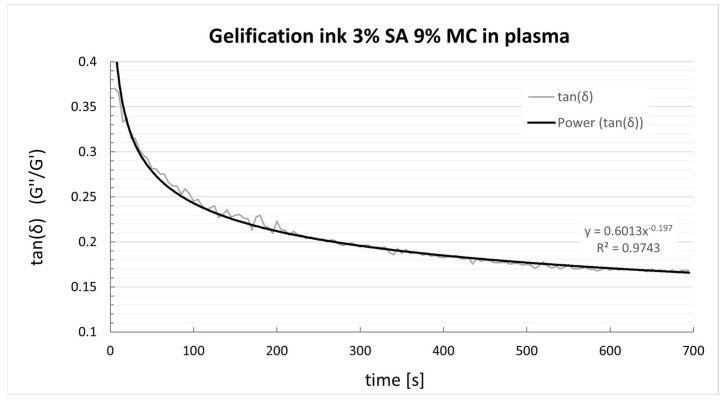
tan(δ) evolution of P-Alg_3_/MC_9_ ink after adding 1.5 mL of CaCl_2_ 1.5%.

The evolution of the tan(δ) after adding the ionic crosslinker is displayed in Figure 6. The ink gels quickly during the first five minutes, then slowly gains elastic prevalence, thus decreasing tan(δ). Past 20 min from the crosslinker solution addition, the constructs have swelled slightly, doubling the filament diameter yet not clogging the printed grids.

**Figure 7:**
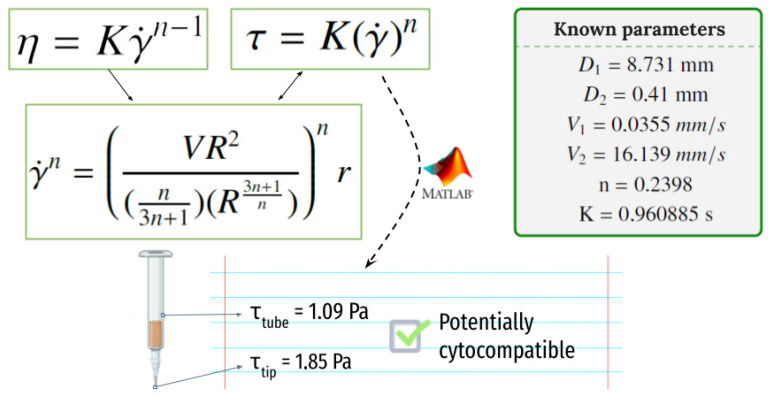
The shear rate that the cells will suffer when printing at the syringe and at the needle tip.

A representation of the syringe used for the process of printing and the shear rate calculations made to evaluate the shear rate that cells will suffer when printing (and thus to evaluate their potential for survival) is shown in Figure 7.

With a printing pressure of 78 ± 1 kPa, the required time to dispense 3 mL of ink has been measured to be 23′28″. Using the equation shown in Figure 7, a V_2_ = 16.139 mm/s speed at the tip of the dispensing needle and a V_1_ = 0.0355 mm/s speed at the core of the syringe have been determined. Equation (2) can now be used to determine the shear rate the cells will suffer when printing and to evaluate the potential for survival in the printing process. Calculated values at the syringe and the tip of the needle are τ_max1_ = 1.088 Pa and τ_max1_ = 1.845 Pa, respectively.

### 3.2. Viability Assays on Cell-Laden Constructs

**Figure 8:**
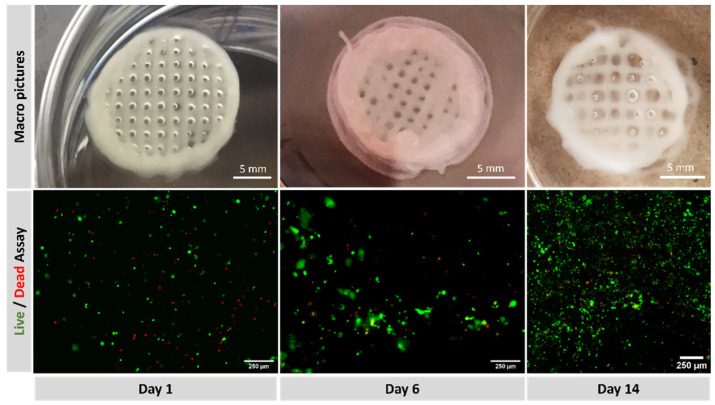
(**Top**): macroscopic view of printed constructs at days 1, 6 and 14. (**Bottom**): fluorescence with green (calcein staining) for live cells and red (EthD-1) for dead, at days 1, 6 and 14.

The top row in Figure 8 displays a macroscopic picture of the printed constructs on days 1, 6 and 14. The image after six days shows that the construct maintains its structural integrity and still can be handled. The image after 14 days shows a swollen construct with diffuse edges indicating that the degradation process has started to affect the integrity of the construct. Figure 8, bottom row, collects the fluorescent channels red and green at days 1 and 6 taken with the Smart Cell Imager PAULA and the live/dead ratio at day 14 taken with a Leica TCS SPE confocal microscopy. Some red dots can be observed on day one, suggesting that the printing process was barely detrimental for cells. Green fluorescence occupies a larger surface at day six, indicating a good proliferation rate after six days of culture. Calculations based on fluorescence image yield cell viability of 74 ± 4% at day 1, 81 ± 4% at day 6 and 77 ± 1 at day 14.

**Figure 9:**
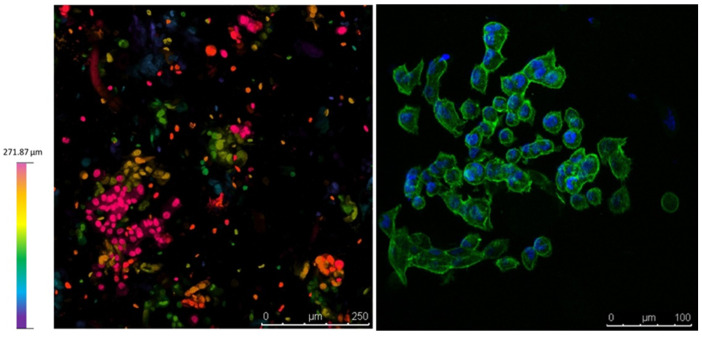
Confocal microscopy on day 9. (**Left**), depth map showing that cells survive at different levels within the construct. (**Right**), Dapi/Actin staining for the nucleus and cytoplasm shows the PANC-1 phenotype within the construct.

Figure 9 collects confocal microscopy images of the constructs. The left image, a depth map, shows how the cells are distributed in different z-levels along the construct, and the colour corresponds to cells at different depths. Cell aggregates for the total length of the cut can be appreciated, suggesting a good proliferation rate to varying heights of the construct. In the right image, a blue DAPI staining for the nucleus and a green actin staining for the cytoplasm enable us to appreciate the formation of cell aggregates and the maintenance of the characteristic prismatic forms of the PANC-1 cells selected.

## 4. Discussion

### 4.1. Selection of Bioink Composition, Printability and Viscoelastic Properties

Plasma would be an ideal vehicle for cell-laden bioinks because it contains over 700 proteins such as fibrinogen, fibronectin, or albumin as significant components and growth factors, hormones, and cytokines that would assist cell growth and survival [23]. Plasma enabled the development of patient-specific bioinks suitable for the bioprinting of constructs with patient-specific shapes, material composition, and cells [23]. This is particularly relevant for cancer modelling since anticancer drugs have been shown to vary substantially among patients [34], and there is a heightened initiative to develop in vitro tumour models that could be patient-derived and able to phenocopy as much as possible from the original tumour [35]. However, plasma consistency is similar to water and requires supplementation to reach the viscoelastic properties needed for bioprinting [36]. Some studies have shown that mixing plasma with polysaccharides such as cellulose nanocrystals or alginate proves the compatibility among these components and shows great potential for building bioinks with regenerative abilities that could be used in personalized medicine [13,37].

Alginate-based bioinks are among the most used and successful materials in bioprinting due to their shear thinning character, the ability for rapid crosslinking, which provides good shape fidelity to the printed construct, and the feasibility of printing viable cells [38]. Since the viscosity of alginate bioinks depends on the alginate concentration, the alginate molecular weight and the cell density loading, printability could be promoted by controlling these parameters and blending with different saccharides. Using different alginate concentrations have been discharged because it may affect cellular viability, as will be discussed later. The addition of methylcellulose (MC) to a low-concentrated alginate solution has previously been shown to strongly improve the printability of alginate solutions, enabling 3D plotting of centimetre-scaled 3D constructs with tailored architecture and with high shape fidelity [24,25]. Based on these arguments, three different plasma-based inks were proposed for this project, low-concentrated alginate (P-Alg_3_) and two inks modified with MC, P-Alg_3_/MC_3_ and P-Alg_3_/MC_9_. Printability was tested with these three inks. The three inks present a shear thinning behaviour that should reduce the shear stress of cells during printing. It can be noticed that inks modified with MC expand the range of shear thinning behaviour compared to the P-Alg_3_/MC_0_ ink, as seen in Figure 2. This would also expand the range of conditions and printing techniques; i.e., inkjet techniques require lower viscosities than extrusion techniques [39], which could be used with these inks, though this is not the subject of this work.

In a first analysis, viscosity during printing and, consequently, the shear stress suffered by cells should be higher in p-Alg_3_/MC_9_ than in the other two inks. However, rheological properties must also be adjusted to generate constructs with high shape fidelity. And, as can be seen in Figure 4, P-Alg_3_/MC_9_ ink is the most stable over the whole range of frequencies, also showing the lower value for tan(δ), indicating the highest elastic behaviour out of the three inks. Thus, the highest capacity for absorbing impacts with no deformation. Also, only P-Alg_3_/MC_9_ was consistent enough to pile layers with no merging, as displayed in Figure 1. Thus it would be the only of the three proposed inks capable of printing multilayer constructs. In addition, P-Alg_3_/MC_9_ is the ink that eventually recovers most of the viscosity that it had before printing (Figure 3). This result indicates that P-Alg_3_/MC_9_ is the ink that suffers lower damage during the printing process and suggests that higher printing fidelity would be achievable. When the ionic crosslinker is added immediately after printing, the printed ink displays a quick gelling behaviour that provides further consistency, as deduced from the quick decrease of the tan(δ) value (Figure 7). Eventually, the quick “solidification” produces an acceptable printing fidelity, as can be observed in Figure 5, comparing the device designed and the device printed. However, even this ink displays some swelling that somehow limits the fidelity of the design and suggests that a minimum separation distance between printed lines would be required.

The suitability of our bioink for modelling pancreas tumours can be considered by comparison with natural tissues. The elastic modulus of diseased tissue is frequently higher than that of healthy tissue. In particular, for the pancreas, human pancreatitis tissue possesses a modulus two times higher than healthy tissue. In contrast, tumour tissues are five times higher than healthy tissue [40]. In rheology experiments, pancreas tissue models have been reported to be in the range from 120 to 180 Pa with a weakly frequency-dependent dynamic modulus from 0.1 to 0.8 Hz [41], whereas the reported values from our bioink are in the range of 400 to 1100 Pa, thus roughly 4 to 6 times higher than the healthier tissue model in the literature.

### 4.2. Crosslinking Behaviour

Only the P-Alg_3_/MC_9_ ink displayed a printing fidelity appropriate for building up 3-dimensional constructs. Thus, only this ink was selected for testing the gelation process and cellular viability. In addition, extrusion bioprinting requires quick gelation of the bioink after the printing process to maintain the structure long enough for the cells to proliferate [42]. Alginate hydrogels offer a rapid gelation ability by ionic crosslinking with divalent cations. The rheology test of P-Alg_3_/MC_9_ showed a reduction of the tanδ value from 0.4 to 0.2 in five minutes after adding CaCl_2,_ followed by a slow stabilization later. The prevalence of the elastic component over the viscous component of the material should help maintain the construct’s morphology for the required period for cell proliferation. (Figure 6). However, it has to be clear that this behaviour is due to the specific characteristics of the selected alginate, and then it is specific to the prepared ink. Ionic crosslinking of alginate is a complex process that depends on the M and G blocks content of the corresponding alginate. G blocks form ionic bridges with divalent cations and increase the gel-forming ability, whereas M blocks form weak junctions with divalent cations, and MG and M blocks increase flexibility [38]. It has to be considered that the biological response depends on this ratio since it has been described that a high amount of M blocks could cause immunogenicity [43]. The crosslinking mechanism involves the coordination of divalent ions with four-carboxyl groups to form an egg-box arrangement, as described in previous work [38]. Our ink was prepared with the alginic acid sodium salt of Mw: 8945 g/mol and a M/G ratio of 0.63. Full characterization of the alginate used is described in a former paper [28], and the elasticity of the printed construct, crosslinking ability and biological response are only reproducible with this alginate Mw and M/G ratios. The batch-to-batch variability reported as one of the problems with 3D models in tissue engineering or cancer research [44] may be related to the variability of the alginates used or the lack of specifications of the polysaccharides used.

### 4.3. PANC-1 Viability on 3D Bioprinted Constructs

As stated above, the printability of alginate-based bioinks can be promoted by modifying the alginate concentration or the alginate molecular weight or by blending with different saccharides. Since low-concentrated alginate solutions have been shown to improve the printability of the material strongly [38] and also high concentrations can negatively influence long-term biological performance [45], it was decided that the incorporation of an additional saccharide to be a more convenient approach to combine printability and cellular viability. The composition P-Alg_3_/MC_9_ is the most convenient prepared ink from the printability side. It is necessary to check whether it is also suitable for our particular cell line survival as well. Firstly, it was calculated that the maximum shear stress at the syringe would be τ_max1_ = 1088 Pa, whereas, at the tip, it would be τ_max1_ = 1845 Pa, and since a conical tip is being used, the cell will suffer this stress for about 2 milliseconds only [46]. However, short-time exposure to high levels of shear stress has been shown that affects cell viability and can induce long-term alterations in the proliferation potential of the cells that have survived the printing process [47,48]. The literature describes that fibroblast viability dropped below 80% for shear stress higher than 5000 Pa for 30 ms [46] and a maximum shear stress value of 160 Pa was detrimental to chondrocyte viability [49]. These results st then that each cell type has a different resistance. Nor and no studies have been found so far about the PANC-1 cell line needed for building pancreatic tumor models and cell viability needs to be checked after printing. Cell morphology, proliferation and phenotype have been examined after printing and after two weeks of printing. Results are collected in Figure 8 and Figure 9. It can be observed that viability is not significantly affected, and phenotypic expressions are preserved [50,51], thus, pending further examination. They are expected to behave similarly as they would in their native niches. Also, good control of the spatial repartition of the cells and cell density can be observed (Figure 9 left), enabling a reproducible cell patterning that can be used to develop a controlled tumour microenvironment.

In summary, the proper selection of the bioprinting parameters, starting with the correct selection of the bioink components, the viscosity achieved, the capacity for printing recovery, and the exposure to the shear stress during the bioprinting process are major challenges that need to be overcome to facilitate any application of 3D bioprinting. Gelling kinetics is the key parameter to obtain constructs that maintain the designed features, and it is also of great importance to preserve the cell bioactivity and phenotypic expressions in the way they behave in their native niches. Our results show for the first time that the combination of a PANC-1/P-alg_3_/MC_9_ bioink and a pneumatic extrusion configuration with a 0.4 mm end conical nozzle can be used to build up a pancreatic tumour 3D biomodel. Although these results are specific only for the characteristics of the alginate selected and for the chosen cell line and thus, they cannot be directly applied to other hydrogels, printing techniques or cell lines, these results are of great value for other biofabrication modelling attempts as they give a tip on which parameters should be examined, how they should be examined and what should be expected from the cells respond.

## 5. Conclusions

Our results show that a PANC-1, plasma-loaded bioink with a 3% alginate concentration and alginate: methylcellulose ratio 1:3 provides a viscosity and printing recovery features suitable for bioprinting. A 3D bioprinting configuration based on pneumatically pressure extrusion with a conical 0.4 mm width end nozzle induces a τmax1 = 1845 Pa low enough to maintain PANC-1 phenotype, viability over 70% and proliferation in suitable levels for building up PANC-1-based 3D printed biomodels. The bioink composition and printing conditions did not inhibit a homogeneous proliferation of PANC-1 cells at different heights, suggesting the viability of building three-dimensional bioprinted pancreas tumor models based on PANC-1/P-Alg_3_/Mc_9_ bioinks.

## Data Availability

The data that support the findings of this study are available on request from the corresponding author.

## References

[1] Gebeyehu A., Surapaneni S.K., Huang J., Mondal A., Wang V.Z., Haruna N.F., Bagde A., Arthur P., Kutlehria S., Patel N. (2021). Polysaccharide Hydrogel Based 3d Printed Tumor Models for Chemotherapeutic Drug Screening. Sci. Rep..

[2] Peela N., Truong D., Saini H., Chu H.H., Mashaghi S., Ham S.L., Singh S., Tavana H., Mosadegh B., Nikkhah M. (2017). Advanced Biomaterials and Microengineering Technologies to Recapitulate the Stepwise Process of Cancer Metastasis. Biomaterials.

[3] Zhang Y.S., Duchamp M., Oklu R., Ellisen L.W., Langer R., Khademhosseini A. (2016). Bioprinting the Cancer Microenvironment. ACS Biomater. Sci. Eng..

[4] Poggi A., Villa F., Fernadez J.L.C., Costa D., Zocchi M.R., Benelli R. (2021). Three-Dimensional Culture Models to Study Innate Anti-Tumor Immune Response: Advantages and Disadvantages. Cancers.

[5] Chaicharoenaudomrung N., Kunhorm P., Noisa P. (2019). Three-Dimensional Cell Culture Systems as an In Vitro Platform for Cancer and Stem Cell Modeling. World J. Stem Cells.

[6] Tian C., Tu Q., Liu W., Wang J. (2019). Recent Advances in Microfluidic Technologies for Organ-on-a-Chip. Trac-Trends Anal. Chem..

[7] Rodrigues T., Kundu B., Silva-Correia J., Kundu S.C., Oliveir J.M., Reis R.L., Correlo V.M. (2018). Emerging Tumor Spheroids Technologies for 3d in Vitro Cancer Modeling. Pharmacol. Ther..

[8] Gunti S., Hoke A.T.K., Vu K.P., London N.R. (2021). Organoid and Spheroid Tumor Models: Techniques and Applications. Cancers.

[9] Vinci M., Gowan S., Boxall F., Patterson L., Zimmermann M., Court W., Lomas C., Mendiola M., Hardisson D., Eccles S.A. (2012). Advances in Establishment and Analysis of Three-Dimensional Tumor Spheroid-Based Functional Assays for Target Validation and Drug Evaluation. BMC Biol..

[10] Loessner D., Stok K.S., Lutolf M.P., Hutmacher D.W., Clements J.A., Rizzi S.C. (2010). Bioengineered 3d Platform to Explore Cell–Ecm Interactions and Drug Resistance of Epithelial Ovarian Cancer Cells. Biomaterials.

[11] Sung K.E., Beebe D.J. (2014). Microfluidic 3d Models of Cancer. Adv. Drug Deliv. Rev..

[12] Nishiguchi A., Matsusaki M., Asano Y., Shimoda H., Akashi M. (2014). Effects of Angiogenic Factors and 3d-Microenvironments on Vascularization within Sandwich Cultures. Biomaterials.

[13] Cubo N., Garcia M., del Cañizo J.F., Velasco D., Jorcano J.L. (2016). 3d Bioprinting of Functional Human Skin: Production and In Vivo Analysis. Biofabrication.

[14] Cortes E.D., Molina C.M., Rodriguez-Lorenzo L., Cubo-Mateo N. (2023). Generation of Controlled Micrometric Fibers inside Printed Scaffolds Using Standard Fdm 3d Printers. Polymers.

[15] Hughes A.M., Kolb A.D., Shupp A.B., Shine K.M., Bussard K.M. (2021). Printing the Pathway Forward in Bone Metastatic Cancer Research: Applications of 3d Engineered Models and Bioprinted Scaffolds to Recapitulate the Bone–Tumor Niche. Cancers.

[16] Pati F., Gantelius J., Svahn H.A. (2016). 3d Bioprinting of Tissue/Organ Models. Angew. Chem. Int. Ed..

[17] Sánchez-Salazar M.G., Álvarez M.M., Santiago G.T.-D. (2021). Advances in 3d Bioprinting for the Biofabrication of Tumor Models. Bioprinting.

[18] Herrada-Manchón H., Celada L., Rodríguez-González D., Fernández M.A., Aguilar E., Chiara M.D. (2021). Three-Dimensional Bioprinted Cancer Models: A Powerful Platform for Investigating Tunneling Nanotube-Like Cell Structures in Complex Microenvironments. Mater. Sci. Eng. C.

[19] Sigaux N., Pourchet L., Breton P., Brosset S., Louvrier A., Marquette C.A. (2019). 3D Bioprinting: Principles, fantasies and prospects. Oral Maxillofac. Surg..

[20] Xu Z., Lam M.T., Rehm B.H.A., Moradali M.F. (2018). Alginate Application for Heart and Cardiovascular Diseases. Alginates and Their Biomedical Applications.

[21] de Vos P., Lazarjani H.A., Poncelet D., Faas M.M. (2014). Polymers in Cell Encapsulation from an Enveloped Cell Perspective. Adv. Drug Deliv. Rev..

[22] Dani S., Ahlfeld T., Albrecht F., Duin S., Kluger P., Lode A., Gelinsky M. (2021). Homogeneous and Reproducible Mixing of Highly Viscous Biomaterial Inks and Cell Suspensions to Create Bioinks. Gels.

[23] Ahlfeld T., Cubo-Mateo N., Cometta S., Guduric V., Vater C., Bernhardt A., Akkineni A.R., Lode A., Gelinsky M. (2020). A Novel Plasma-Based Bioink Stimulates Cell Proliferation and Differentiation in Bioprinted, Mineralized Constructs. ACS Appl. Mater. Interfaces.

[24] Ahlfeld T., Guduric V., Duin S., Akkineni A.R., Schütz K., Kilian D., Emmermacher J., Cubo-Mateo N., Dani S., Witzleben M.V. (2020). Methylcellulose-a Versatile Printing Material That Enables Biofabrication of Tissue Equivalents with High Shape Fidelity. Biomater. Sci..

[25] Schütz K., Placht A.-M., Paul B., Brüggemeier S., Gelinsky M., Lode A. (2017). Three-Dimensional Plotting of a Cell-Laden Alginate/Methylcellulose Blend: Towards Biofabrication of Tissue Engineering Constructs with Clinically Relevant Dimensions. J. Tissue Eng. Regen. Med..

[26] Rasheed Z.A., Matsui W., Maitra A., Munshi H.G., Grippo P.J. (2012). Pathology of Pancreatic Stroma in Pdac. Pancreatic Cancer and Tumor Microenvironment.

[27] Gonzalez I., Luzuriaga J., Valdivieso A., Candil M., Frutos J., Lopez J., Hernandez L., Rodriguez-Lorenzo L., Yaguee V., Blanco J.L. (2023). Low-Intensity Continuous Ultrasound to Inhibit Cancer Cell Migration. Front. Cell Dev. Biol..

[28] Hernández-González A.C., Téllez-Jurado L., Rodríguez-Lorenzo L.M. (2020). Synthesis of in-Situ Silica-Alginate Hybrid Hydrogels by a Sol-Gel Route. Carbohydr. Polym..

[29] Chernecky C.C., Berger B.J. (2013). Laboratory Tests & Diagnostic Procedures.

[30] Deer E.L., Gonzalez-Hernandez J., Coursen J.D., Shea J.E., Ngatia J., Scaife C.L., Firpo M.A., Mulvihill S.J. (2010). Phenotype and Genotype of Pancreatic Cancer Cell Lines. Pancreas.

[31] Cukjati D., Rebersek S., Miklavcic D. (2001). A Reliable Method of Determining Wound Healing Rate. Med. Biol. Eng. Comput..

[32] Martínez Ávila H., Schwarz S., Rotter N., Gatenholm P. (2016). 3d Bioprinting of Human Chondrocyte-Laden Nanocellulose Hydrogels for Patient-Specific Auricular Cartilage Regeneration. Bioprinting.

[33] Li H., Liu S., Li L. (2016). Rheological Study on 3d Printability of Alginate Hydrogel and Effect of Graphene Oxide. Int. J. Bioprint..

[34] Verma M. (2012). Personalized Medicine and Cancer. J. Pers. Med..

[35] Rizzo G., Bertotti A., Leto S.M., Vetrano S. (2021). Patient-Derived Tumor Models: A More Suitable Tool for Pre-Clinical Studies in Colorectal Cancer. J. Exp. Clin. Cancer Res..

[36] Li M., Tian X., Kozinski J.A., Chen X., Hwang D.K. (2015). Modeling Mechanical Cell Damage in the Bioprinting Process Employing a Conical Needle. J. Mech. Med. Biol..

[37] Mendes B.B., Gómez-Florit M., Hamilton A.G., Detamore M.S., Domingues R.M.A., Reis R.L., Gomes M.E. (2019). Human Platelet Lysate-Based Nanocomposite Bioink for Bioprinting Hierarchical Fibrillar Structures. Biofabrication.

[38] Hernández-González A.C., Téllez-Jurado L., Rodríguez-Lorenzo L.M. (2020). Alginate Hydrogels for Bone Tissue Engineering, from Injectables to Bioprinting: A Review. Carbohydr. Polym..

[39] Hölzl K., Lin S., Tytgat L., Van Vlierberghe S., Gu L., Ovsianikov A. (2016). Bioink Properties before, During and after 3d Bioprinting. Biofabrication.

[40] Rubiano A., Delitto D., Han S., Gerber M., Galitz C., Trevino J., Thomas R.M., Hughes S.J., Simmons C.S. (2018). Viscoelastic Properties of Human Pancreatic Tumors and in Vitro Constructs to Mimic Mechanical Properties. Acta Biomater..

[41] Nicolle S., Noguer L., Palierne J.F. (2013). Shear Mechanical Properties of the Porcine Pancreas: Experiment and Analytical Modelling. J. Mech. Behav. Biomed. Mater..

[42] Axpe E., Oyen M.L. (2016). Applications of Alginate-Based Bioinks in 3d Bioprinting. Int. J. Mol. Sci..

[43] Kulseng B., Skjak-Braek G., Ryan L., Andersson A., King A., Faxvaag A., Espevik T. (1999). Transplantation of Alginate Microcapsules-Generation of Antibodies against Alginates and Encapsulated Porcine Islet-Like Cell Clusters. Transplantation.

[44] Ashworth J.C., Thompson J.L., James J.R., Slater C.E., Pijuan-Galitó S., Lis-Slimak K., Holley R.J., Meade K.A., Thompson A., Arkill K.P. (2020). Peptide Gels of Fully-Defined Composition and Mechanics for Probing Cell-Cell and Cell-Matrix Interactions In Vitro. Matrix Biol..

[45] Freeman F.E., Kelly D.J. (2017). Tuning Alginate Bioink Stiffness and Composition for Controlled Growth Factor Delivery and to Spatially Direct Msc Fate within Bioprinted Tissues. Sci. Rep..

[46] Lucas L., Aravind A., Emma P., Christophe M., Edwin-Joffrey C. (2021). Rheology, Simulation and Data Analysis toward Bioprinting Cell Viability Awareness. Bioprinting.

[47] Blaeser A., Campos D.F.D., Puster U., Richtering W., Stevens M.M., Fischer H. (2016). Controlling Shear Stress in 3d Bioprinting Is a Key Factor to Balance Printing Resolution and Stem Cell Integrity. Adv. Healthc. Mater..

[48] Cidonio G., Glinka M., Dawson J.I., Oreffo R.O.C. (2019). The Cell in the Ink: Improving Biofabrication by Printing Stem Cells for Skeletal Regenerative Medicine. Biomaterials.

[49] Müller M., Öztürk E., Arlov Ø., Gatenholm P., Zenobi-Wong M. (2017). Alginate Sulfate–Nanocellulose Bioinks for Cartilage Bioprinting Applications. Ann. Biomed. Eng..

[50] Longati P., Jia X., Eimer J., Wagman A., Witt M.-R., Rehnmark S., Verbeke C., Toftgård R., Löhr M., Heuchel R.L. (2013). 3D Pancreatic Carcinoma Spheroids Induce a Matrix-Rich, Chemoresistant Phenotype Offering a Better Model for Drug Testing. BMC Cancer.

[51] Gorchs L., Ahmed S., Mayer C., Knauf A., Moro C.F., Svensson M., Heuchel R., Rangelova E., Bergman P., Kaipe H. (2020). The Vitamin D Analogue Calcipotriol Promotes an Anti-Tumorigenic Phenotype of Human Pancreatic Cafs but Reduces T Cell Mediated Immunity. Sci. Rep..

